# Prevalence and Risk Factors Associated with Hepatitis B and Hepatitis C Infections among Patients Undergoing Hemodialysis: A Single-Centre Study in Somalia

**DOI:** 10.1155/2021/1555775

**Published:** 2021-11-13

**Authors:** Mohamed Osman Omar Jeele, Rukia Omar Barei Addow, Faduma Nur Adan, Liban Hassan Jimale

**Affiliations:** ^1^Mogadishu Somali Turkish Training and Research Hospital, Mogadishu, Somalia; ^2^Jazeera University Hospital, Mogadishu, Somalia

## Abstract

**Introduction:**

Hemodialysis patients have the highest risk for developing hepatitis B virus (HBV) and hepatitis C virus (HCV) than the general population. There is no study available for HBV and HCV in this population in Somalia. The main objective of this study is to determine the prevalence and risk factors of HBV and HCV infections among hemodialysis patients in Somalia.

**Methods:**

A cross-sectional assessment of hemodialysis patients from January 2021 to June 2021 was used in this study. 220 patients were included in this study. Age, sex, duration of hemodialysis, number of hemodialysis sessions per week, history of blood transfusion, HbsAg, and anti-HCV antibodies were examined.

**Results:**

Out of the 220 patients, males were predominant (113 (51.4%)). The mean age of the participants was 52.70. The prevalence of HBV was 7.3% (16 respondents), while the prevalence of HCV was 3.2% (7 respondents). 1 respondent (0.5%) had both HBV and HCV. There is a positive correlation between the duration of hemodialysis and the prevalence of HBV and HCV (*r*(218) = 0.298, *p* value <0.001), blood transfusion and prevalence of HBV and HCV (*r*(218) = 0.347, *p* value <0.001), and the number of hemodialysis sessions per week and prevalence of HBV and HCV (*r*(218) = 0.402, *p* value <0.001). The regression model of the combined predictors of history of blood transfusion, duration of hemodialysis, and number of dialysis sessions per week is *R*^2^ = 0.25, which indicates a 25% variance in the prevalence of HBV and HCV with a significance of *F* (3,216) = 23.67, *p* < 0.001.

**Conclusions:**

The prevalence of HBV and HCV among hemodialysis patients in this study was 7.3% and 3.2%, respectively. 0.5% of the respondents had both HBV and HCV. History of blood transfusion, duration of hemodialysis, and number of hemodialysis sessions per week appear to have a strong correlation with the prevalence of HBV and HCV.

## 1. Introduction

Hepatitis B virus (HBV) is a serious worldwide public health problem as 391 million people, or 5% of the world's population, had chronic hepatitis B infection as of 2017, while another 145 million cases of acute HBV infection occurred that year alone [1]. Africa has the highest regional prevalence of hepatitis B (7%) compared to America (0.7%) [2]. Although there is a vaccine for HBV, it remains a global health problem. HBV infection can be acute and later become chronic, leading to end-stage liver disease and eventually to cirrhosis and hepatocellular carcinoma [3]. Hepatitis C virus (HCV) in its terms is a global burden as WHO stated in the 2017 global hepatitis report that 71 million people, approximately 1% of the global population, lived with hepatitis C virus [4]. HCV primarily spreads through contacting contaminated body fluids by blood-to-blood contact associated with injection drug use, poorly sterilized medical equipment, sexual contact, and needlestick injuries in healthcare [5]. It can be also transmitted by blood transfusion although it is less than one case per 2 million blood transfusions [6]. HBV and HCV are the most common viruses in hemodialysis population [7]. Hemodialysis patients have the highest risk for developing hepatitis B virus (HBV) and hepatitis C virus (HCV) than the general population [8]. The hemodialysis process includes removing blood from the patient with needles and plastic tubing, and then blood is pumped into the dialysis membrane. Poisons and toxins cross the dialysis membrane into the dialysate, which discards the harmful substances, and then the blood is returned to the patient [9]. The number of end-stage renal disease patients requiring hemodialysis is increasing steadily over the last decades [10, 11]. Although renal transplantation offers improved quality of life and increases survival rate compared to hemodialysis, hemodialysis is still the choice of renal replacement therapy for end-stage renal disease patients in low-income countries such as Somalia [12, 13]. The prevalence of chronic kidney disease in East Africa is thought to be 14.4% [14], but there is no single study that covers the prevalence of chronic kidney disease specifically in Somalia or the prevalence of hemodialysis patients in Somalia. Around the world, the prevalence of hepatitis B and hepatitis C viruses among hemodialysis patients varies among the countries [7]. Since there is no previous study regarding this subject made in Somalia, this study is the first of its kind to be done in Somalia and intends to be the building block of many studies to be done in Somalia over this subject. This study intends to answer if there is a relationship between the occurrence of HBV and HCV infections and hemodialysis duration or hemodialysis sessions per week or history of blood transfusions. The main objective of this study is to determine the prevalence and risk factors associated with HBV and HCV infections among hemodialysis patients.

## 2. Methods

### 2.1. Aim of the Study

The goal of the study is to determine the prevalence and risk factors associated with HBV and HCV infections among hemodialysis patients in Somalia.

### 2.2. Study Population and Design

This study is a cross-sectional study of hemodialysis patients from January 2021 to June 2021 who are routinely undergoing hemodialysis in Mogadishu Somali Turkish Training and Research Hospital.

The patients' age, sex, and diagnosis were extracted from the hospital database using Hospital Information System (HIS). Other variables such as duration of hemodialysis, number of hemodialysis sessions per week, and history of blood transfusion were collected from the patients through a multiple choice questionnaire. The questionnaire was taken by one of our authors to strength the accuracy of the data. Age, sex, duration of hemodialysis, number of hemodialysis sessions per week, history of blood transfusion, HbsAg, and anti-HCV antibodies were the examined variables.

In southern Somalia, there are only three hemodialysis centers which are located in the capital city of Mogadishu that serves hemodialysis patients. Mogadishu Somali Turkish Training and Research Hospital is the only tertiary hospital among these three hemodialysis centers and has served 300 hemodialysis patients through morning, noon, afternoon, and evening shifts.

The hospital has 30 hemodialysis machines, and 28 of them are used for non-HBV and non-HCV positive patients. One of the remaining two hemodialysis machines is used for HBV positive patients, and the other one is used for HCV positive patients. The hospital does not reuse the hemodialysis membrane, so every dialyzer is a one-time use. After every hemodialysis session, the hemodialysis machine is sterilized with 50% citric acid liquid for 30 minutes before the next patient's session begins. Patients with HBV and HCV always take their hemodialysis sessions in separate rooms from non-HBV and non-HCV patients.

Most of the patients have one or two sessions of hemodialysis per week. This is due to the fact that the government is not covering the cost of hemodialysis patients, so every patient pays from his pocket. The other two hospitals have a total of 200 hemodialysis patients who are going to hemodialysis routinely.

Because of this, we used Raosoft website for determining the sample size. We assumed the total number of hemodialysis patients to be 500, and with 5% margin of error and a confidence level of 95%, a sample size of 220 patients was determined. Patients who were going to hemodialysis in less than 90 days and patients who were unwilling to participate were excluded from this study.

### 2.3. Laboratory Technique

5 ml of blood were drawn from every participant before the hemodialysis session. The samples were centrifuged for 5 minutes at a rate of 3000 rpm, and then serological qualitative tests for HbsAg and anti-HCV antibodies were performed using enzyme-linked immunosorbent assay (ELISA) through a VITROS 3600 machine (Germany). The hospital did not have the capacity to test HBV and HCV genetic materials, and for that reason, we were unable to obtain that data.

### 2.4. Ethical Consideration

Informed consent was obtained from the patients. No personal data have been revealed in this study. The study had been evaluated and approved by the Mogadishu Somali Turkish Training and Research Hospital ethical committee.

### 2.5. Statistical Analysis

The data were collected and analyzed using Statistical Package for Social Sciences (SPSS) software version 26. Descriptive statistics, compare mean, Pearson correlations, and multiple regression were used in this study.

## 3. Results

Out of the 220 patients that were enrolled in the study, 113 (51.4%) were males and 107 (48.6%) were females. The mean age of the participants was 52.70 with maximum and minimum age ranging between 11 years and 88 years (Table 1). Among the 220 patients, 111 (50.4%) have been undergoing hemodialysis between 12 months and 36 months.

142 (64.5%) of our respondents had 2 sessions of hemodialysis per week, 60 (27.3%) respondents had once-a-week sessions, and 18 (8.2%) had a frequency of 3 sessions of hemodialysis per week (Table 2).

**Figure 1:**
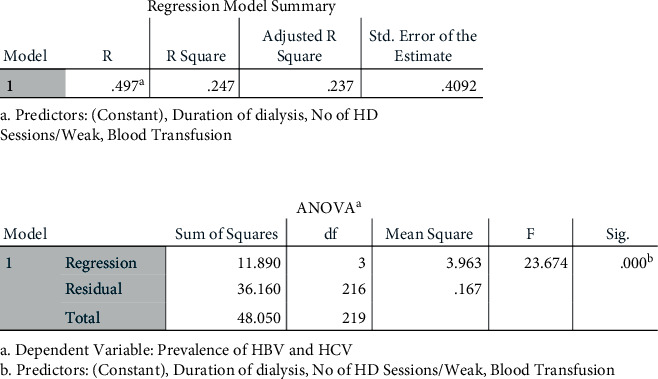
The regression model and its significance of the predictors towards the prevalence of HBV and HCV among hemodialysis patients.

In this study, the prevalence of HBV was 7.3% (16 respondents) and the prevalence of HCV was 3.2% (7 respondents). Only 1 respondent (0.5%) is positive for both HBV and HCV (Table 3). The data also showed that, among the 16 respondents that were tested positive for HBV, two-thirds of the respondents (11 (68.75%)) were males while females comprised the remaining 5 respondents (31.25%). Among the age subgroups, the prevalence of HBV was 0%, 18.75%, 25%, 50%, and 6.25% in less than 20 years, 21–40 years, 41–60 years, 61–80 years, and more than 80 years, respectively. Of the 16 respondents who were HBV positive, 11 (68.75%) had been on hemodialysis for more than 36 months while the remaining 5 respondents (31.25%) have been on hemodialysis for 12–36 months. Out of the 7 respondents who were tested positive for HCV, 4 respondents (57%) were males and 3 respondents (43%) were females. Among the age subgroups, the prevalence of HCV was 14.3%, 0%, 28.6%, 57.1%, and 0% in less than 20 years, 21–40 years, 41–60 years, 61–80 years, and more than 80 years, respectively. Of the 7 respondents who were tested positive for HCV, 5 (71.4%) had more than 36 months of hemodialysis (Table 2). The majority of respondents (196 (89.1%)) were seronegative for HBV and HCV (Table 3). The study found that there is a significant positive correlation between the duration of hemodialysis and the prevalence of HBV and HCV (*r*(218) = 0.298, *p* value <0.001). There is also a positive correlation between blood transfusion and the prevalence of HBV and HCV among hemodialysis patients (*r*(218) = 0.347, *p* value <0.001). The study also found a highly significant positive correlation between the number of hemodialysis sessions per week and the prevalence of HBV and HCV (*r*(218) = 0.402, *p* value <0.001) (Table 4). The regression model of the combined predictors of history of blood transfusion, duration of dialysis, and the number of dialysis sessions per week is *R*^2^ = 0.25, which indicates that they have a 25% variance in the prevalence of HBV and HCV among hemodialysis patients with a significance of *F* (3,216) = 23.67, *p* < 0.001 (Figure 1).

## 4. Discussion

Hepatitis B (HBV) and hepatitis C (HCV) are major causes of chronic liver inflammation and thus increase the morbidity and mortality of the patients [15]. Reduced immunity from chronic renal disease makes patients susceptible to succumb HBV and HCV infections [16]. Exposure to multiple blood transfusion and deficient immune response puts the patients with end-stage renal disease (ESRD) at increased risk of acquiring HBV and HCV infections than the general population [7].

A meta-analysis done by Hassan-Kadle et al. in 2018 found the prevalence of HBV infection in Somali population to be 18.9% with the 20–39 age group having the highest prevalence of around 12.4%. In addition, they also found the prevalence of HCV to be around 4.84% and 29.82% of them had already developed chronic liver disease [17].

Long-standing civil war, lack of centralized government, lack of laboratory techniques, and undertrained health workers contributed to the lack of data in many subgroup populations in Somalia [18].

In this study, we revealed the prevalence of HBV among hemodialysis patients to be 7.3% and the prevalence of HCV among hemodialysis patients to be 3.2%. HBV was more common in males and in the age group of 61–80 years. Similarly, HCV was more common in males and in the age group of 61–80 years. We also found that there is a significant positive correlation between the prevalence of HBV and HCV and the history of blood transfusion, the duration of hemodialysis, and the number of hemodialysis sessions per week.

A study done by Almezgagi et al. about the prevalence of HBV and HCV and associated risk factors among hemodialysis patients in Ibb City, Yemen, in 2020 concluded that the prevalence of HBV is 3%, HCV is 21%, and coinfection 2% [19]. This conclusion is different from our study because we found that HBV was more common than HCV among hemodialysis patients in Somalia.

A similar study done in Khartoum, Sudan, by Gasim et al. in 2012 found HCV more common than HBV in 8.5%–4.5%, respectively. In contrast to our study, they also concluded that blood transfusion had no significant association with the prevalence of HBV and HCV [20].

A meta-analysis done by Ashkani-Esfahani et al. regarding the prevalence of hepatitis C virus infection among hemodialysis patients in the Middle East in 2017 reported 25.3% of the overall prevalence of HCV [21]. İn contrast to that, we found that the prevalence of HCV is 3.2%.

Furthermore, a meta-analysis of combined North Africa and Middle East done by Harfouche et al. about the prevalence of HCV among hemodialysis patients in 2017 had a result of 29.2% prevalence rate for HCV [22].

A study done in Lebanon by Rached et al. concluded that the prevalence of HBV and HCV is 1.6% and 4.7%, respectively [23]. This also different from our conclusion that HBV was more common than HCV among hemodialysis patients in Somalia.

The higher prevalence of HBV than HCV in our study can be explained by the higher incidence of HBV among Somali population than HCV as reported by Hassan-Kadle et al. in 2018 [17].

Finally, this study intends to be the gateway of many researchers to look into this subject and for policy makers to implement good public health strategies to tackle this growing public health issue.

One of our study limitations is the absence of data of HBV and HCV testing at the molecular level because there may be cases of hidden HBV that could not be detected through the ELISA test. The other limitation is that there is no specific number of the prevalence of hemodialysis patients in Somalia. This is due to lack of previous study publication regarding this subject.

## 5. Conclusion

The prevalence of HBV and HCV among hemodialysis patients in this study was 7.3% and 3.2%, respectively. 0.5% of the respondents had both HBV and HCV. History of blood transfusion, duration of hemodialysis, and number of hemodialysis sessions per week appear to have a strong correlation with the prevalence of HBV and HCV. Implementation of good infectious control plans, patient education, immunizing patients against HBV, and minimizing the patients' need of blood transfusion by close follow-up can be taken to lower the chance of getting HBV and HCV infections. Upon working on this study, the authors clearly saw firsthand the need to know the prevalence of hemodialysis patients in Somalia, so we also urge other researchers to immediately focus on that subject. The study only focused on a single tertiary hospital in Somalia, so a nation-wide study is needed to determine the prevalence of HBV and HCV among this group of population in Somalia.

## Figures and Tables

**Table 1:** The distribution of age and gender among respondents.

*Age*		
Mean	52.70	
Median	55.00	
Std. deviation	18.479	
Minimum	11	
Maximum	88	

*Gender*		
	Frequency	Percentage
Male	113	51.4%
Female	107	48.6%
Total	220	100%

**Table 2:** The prevalence of HBV and HCV in relation to demographic variables of the respondents.

Demographic variables		Negative	Hepatitis B positive	Hepatitis C positive	Both hepatitis B and C positive	Total
Age group	<20 years	13 (6.6%)	0 (0%)	1 (14.3%)	0 (0%)	14 (6.4%)
	21–40 years	35 (17.9%)	3 (18.75%)	0 (0%)	0 (0%)	38 (17.2%)
	41–60 years	72 (36.7%)	4 (25%)	2 (28.6%)	1 (100%)	79 (36%)
	61–80 years	76 (38.8%)	8 (50%)	4 (57.1%)	0 (0%)	88 (40%)
	>80 years	0 (0%)	1 (6.25%)	0 (0%)	0 (0%)	1 (0.4%)
	Total	196 (100%)	16 (100%)	7 (100%)	1 (100%)	220 (100%)
Gender	Male	97 (49.5%)	11 (68.7%)	4 (57%)	1 (100%)	113 (51.4%)
	Female	99 (50.5)	5 (31.3%)	3 (43%)	0 (0%)	107 (48.6%)
	Total	196 (100%)	16 (100%)	7 (100%)	1 (100%)	220 (100%)
Duration of dialysis	Less than 12 months	52 (26.5%)	0 (0%)	1 (14.3%)	0 (0%)	53 (24.1%)
	12–36 months	105 (53.5%)	5 (31.25%)	1 (14.3%)	0 (0%)	111 (50.4%)
	More than 36 months	39 (20%)	11 (68.75%)	5 (71.4%)	1 (100%)	56 (25.5%)
	Total	196 (100%)	16 (100%)	7 (100%)	1 (100%)	220 (100%)
No. of HD sessions/week	1 session/week	60 (30.6%)	0 (0%)	0 (0%)	0 (0%)	60 (27.3%)
	2 sessions/week	131 (66.8%)	6 (37.5%)	5 (71%)	0 (0%)	142 (64.5%)
	3 sessions/week	5 (2.6%)	10 (62.5%)	2 (29%)	1 (100%)	18 (8.2%)
	Total	196 (100%)	16 (100%)	7 (100%)	1 (100%)	220 (100%)
Blood transfusion	No blood transfusion	53 (27.04%)	1 (6.25%)	2 (28.6%)	0 (0%)	56 (25.5%)
	Less than 5 times transfused	83 (42.3%)	1 (6.25%)	1 (14.3%)	0 (0%)	85 (38.6%)
	5–10 times transfused	42 (21.42%)	6 (37.5%)	1 (14.3%)	0 (0%)	49 (22.2%)
	10–15 times transfused	17 (8.7%)	6 (37.5%)	1 (14.3%)	0 (0%)	24 (11%)
	More than 15 times transfused	1 (0.5%)	2 (12.5%)	2 (28.6%)	1 (100%)	6 (2.7%)
	Total	196 (100%)	16 (100%)	7 (100%)	1 (100%)	220 (100%)

**Table 3:** The prevalence of HBV and HCV among hemodialysis patients in Somalia.

Serological results	Frequency	Percent
Negative	196	89.1
Hepatitis B positive	16	7.3
Hepatitis C positive	7	3.2
Both hepatitis B and C positive	1	0.5
Total	220	100.0

**Table 4:** The correlations between the prevalence of HBV and HCV and the number of hemodialysis sessions/week, blood transfusion, and duration of dialysis.

Prevalence of HBV and HCV		
No. of HD sessions per week	Pearson correlation	0.402^*∗∗*^
	Sig. (2-tailed)	0.000
	*N*	220
Blood transfusion	Pearson correlation	0.347^*∗∗*^
	Sig. (2-tailed)	0.000
	*N*	220
Duration of dialysis	Pearson correlation	0.298^*∗∗*^
	Sig. (2-tailed)	0.000
	*N*	220

^*∗∗*^means there is a correlation between the variables.

## Data Availability

The data are available from the corresponding author upon request.

## Abbreviations

Anti-HCV: Anti-hepatitis C virus antibody
ELISA: Enzyme-linked immunosorbent assay
HBV: Hepatitis B virus
HCV: Hepatitis C virus
HbsAg: Hepatitis B surface antigen
Ml: Milliliter
RPM: Round per minute
WHO: World Health Organization
SPSS: Statistical package for social sciences.
